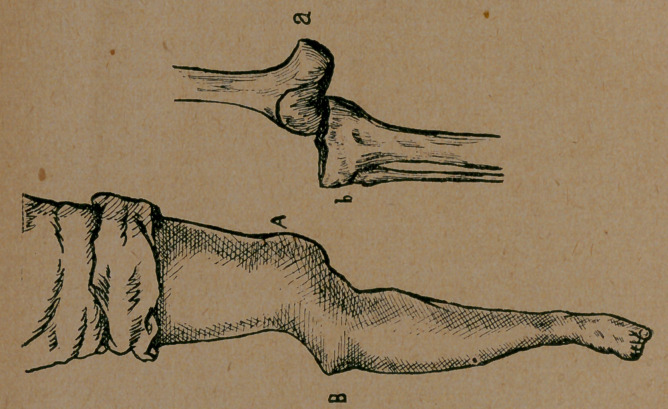# Report of a Case of Outward Dislocation of the Knee

**Published:** 1894-04

**Authors:** T. J. Bennett

**Affiliations:** Austin, Texas


For Texas Medical Journal.

### REPORT OF A CASE OF OUTWARD DISLOCATION OF KNEE.

BY T. J. BENNETT, M. D., AUSTIN, TEXAS.

ROBERT P., age 14, was brought into the office January 24, 1893, suffering from what afterwards proved to be an outward dislocation of the right knee, with a slight fracture of the external tuberosity of the tibial head. The injury was caused by the leg becoming entangled between the wheel and body of a heavily loaded wagon. Just how the force was applied, the boy could not state, and no one saw him when the accident occurred.

The integument was only slightly bruised, and that on the external aspect of the limb opposite the knee joint. Chloroform was administered by Dr. Hudson, and after considerable extension and manipulation reduction was accomplished. It was then ascertained that the external tuberosity of the tibia, including a small portion of the articular surface, had been crushed, and had interfered with the ready reduction of the joint. The limb was put up straight, but the patient experienced so much pain that the splint was removed the next day, and the leg allowed to assume the easiest position, which was at an angle of about forty-five degrees. It was then supported by sand bags, and bathed with cold water and liniments. The swelling of the leg about the knee was very great, and the patient suffered with intense paroxysms of pain. At the end of five days, fluctuation was detected at the point where the pressure had been exerted by the dislocated joint. A bistoury was here inserted, and about two ounces of a dark mixture of blood and pus flowed out. The discharge gradually assumed a purulent character, and from the history continued to flow, though in small quantity, for five months, during which time the patient was lost sight of. It was learned that the patient had been able to walk by the aid of a crutch, though the limb, when examined at this time, was not straight by twenty-five degrees, and the opening on the external side of the knee was discharging a pus indicative of necrosed bone. An incision was made at this point, extending through the old opening and down to the articular junction of the femur and tibia, when a number of small pieces of bone were found and removed. All necrotic tissue was curetted away, and the wound dressed antiseptically. There was no further trouble. The wound healed promptly, the boy gained complete use of his limb, and altogether there was a happy result.

Wyeth (Text-Book of Surgery) says that "traumatic luxation of the knee is comparatively rare," and that the "prognosis after this injury is unfavorable."

## Figures and Tables

**Figure f1:**